# Geographic Variation in Medicare Fee-for-Service Health Care Expenditures Before and After the Passage of the Affordable Care Act

**DOI:** 10.1001/jamahealthforum.2021.4122

**Published:** 2021-12-10

**Authors:** Neeraj Sood, Zhiyou Yang, Peter Huckfeldt, José Escarce, Ioana Popescu, Teryl Nuckols

**Affiliations:** 1 Sol Price School of Public Policy, University of Southern California, Los Angeles; 2 Health Policy Research Center, Mongan Institute, Massachusetts General Hospital, Boston; 3 Division of Health Policy and Management, University of Minnesota School of Public Health, Minneapolis; 4 Division of General Internal Medicine and Health Services Research, Department of Medicine, David Geffen School of Medicine at UCLA (University of California, Los Angeles); 5 Department of Health Policy and Management, UCLA Fielding School of Public Health; 6 Division of General Internal Medicine, Department of Medicine, Cedars-Sinai Medical Center, Los Angeles, California

## Abstract

**Importance:**

Geographic variation in Medicare spending is often used as a measure of wasteful spending. A 2013 Institute of Medicine report found that postacute care was a key contributor of geographic variation from 2007 to 2009. However, payment reforms and antifraud efforts implemented after the passage of the Affordable Care Act (ACA) may have reduced geographic variation in spending, especially postacute care spending.

**Objective:**

To investigate how geographic variation in Medicare fee-for-service per-beneficiary spending changed from 2007 to 2018 before and after passage of the ACA.

**Design, Setting, and Participants:**

This cross-sectional study included all fee-for-service Medicare enrollees 65 years or older from January 1, 2007, to December 31, 2018. The fee-for-service Medicare Geographic Variation Public Use File was used to group hospital referral regions (HRRs) in each year into deciles (10 equal groups) based on per-beneficiary total spending. The difference between the per-beneficiary monthly spending in each decile and the national mean, as well as the ratio of per-beneficiary total spending in the top deciles to that of the bottom decile, were reported. Data analysis occurred from July 22, 2019, to October 21, 2021.

**Main Outcomes and Measures:**

Per-beneficiary spending on hospital inpatient, hospital outpatient, physician, and postacute care (and type of postacute care).

**Results:**

There were 27.2 million fee-for-service beneficiaries in 2007 (58.0% women) and 28.3 million beneficiaries in 2018 (55.9% women). Per-beneficiary Medicare spending was $9691 in 2007 and $9847 in 2018 (using inflation-adjusted 2018 dollars). Geographic variation in Medicare spending was stable from 2007 to 2011 and then declined steadily from 2012 to 2018. The ratio of per-beneficiary total Medicare spending in the HRRs in the top decile to the bottom decile was 1.68 in 2007 ($415 monthly difference in spending) but only 1.56 ($361 monthly difference in spending) in 2018 (estimated change, −0.12 [95% CI, −0.21 to −0.02]; *P* = .01). Focusing on specific spending categories, the only statistically significant reductions in geographic variation were found for home health; the ratio of home health spending among HRRs in the top to bottom deciles of total Medicare spending fell from 5.14 in 2007 to 3.45 in 2018 (change, −1.69 [95% CI, −3.30 to −0.09]; *P* = .04).

**Conclusions and Relevance:**

Geographic variation in total per-beneficiary Medicare spending fell from 2007 to 2018, with home health spending being a key factor associated with geographic variation. The ACA’s value-based payment programs and enhanced integrity efforts in home health provide a possible explanation for the decrease.

## Key Points

**Question:**

Which categories of spending were associated with reductions in geographic variation of Medicare per-beneficiary spending across the US after the passage of the Affordable Care Act?

**Findings:**

In this cross-sectional study of Medicare enrollees aged 65 years or older, geographic variation in Medicare fee-for-service spending per beneficiary was stable from 2007 to 2011 and then declined steadily from 2012 to 2018. A key factor associated with reduced geographic variation in spending was reduced variation in postacute care spending, specifically home health spending.

**Meaning:**

These findings suggest that antifraud enforcement efforts and payment reforms that were instituted as part of the Affordable Care Act may have reduced geographic variation in Medicare fee-for-service per-beneficiary spending, although significant geographic variation remains.

## Teaser

This cross-sectional study explores which components of Medicare spending were associated with reductions in geographic variation and potential policy mechanisms after passage of the Affordable Care Act among enrollees 65 years or older.

## Introduction

In the fee-for-service Medicare system, health care expenditures have been rising for decades and, historically, spending has varied widely across geographic areas.^1,2^ Unexplained geographic variation in health care expenditures has been interpreted as an indicator of wasteful spending.^3^ For example, an Institute of Medicine report found that variation in per-beneficiary monthly spending was not explained by differences in the frequency and severity of health conditions across geographic areas but rather by differences in the practice patterns of clinicians or health care institutions.^4^ Geographic variation in health care may not always represent inappropriate use—areas of lower use may provide below-optimal levels of service—but the variations identified in the Institute of Medicine report had no systematic association with quality of care.^4,5,6^

The Affordable Care Act (ACA), passed on March 23, 2010, established several reforms designed to reduce or limit growth in health care expenditures in fee-for-service Medicare while maintaining or improving quality of care and health outcomes. These reforms included programs such as the Shared Savings Accountable Care Organization program and the Hospital Readmissions Reduction Program.^7^ In addition, the ACA empowered the Centers for Medicare & Medicaid Services (CMS) to more forcefully identify and prevent inappropriate or fraudulent payment by establishing a fraud prevention initiative that allowed Medicare to impose moratoria on enrolling new clinicians in a particular geographic area and expanding the enforcement powers of the Office of the Inspector General.^8^ One area of particular focus of antifraud enforcement has been home health care. Starting in 2009, the US Departments of Health and Human Services and Justice partnered to form health care fraud strike force teams that coordinated federal, state, and local law enforcement to investigate and prosecute Medicaid and Medicare fraud in selected areas of the US.^9^ These efforts led to 350 criminal and civil actions against individuals engaged in home health fraud in the immediate post-ACA period (fiscal years 2011-2015).^10^ In addition, the CMS enacted moratoria on new home health care agencies in geographic areas in Florida, Illinois, Michigan, and Texas starting in 2013.^10^

To the extent that Medicare payment reforms and antifraud efforts disproportionately affected geographic markets that were outliers in terms of health care use and Medicare spending, the ACA could have reduced geographic variation in Medicare spending. There is evidence that growth in per-beneficiary fee-for-service Medicare expenditures slowed nationally after the ACA.^11,12^ Prior work has also shown that geographic variation in fee-for-service Medicare spending narrowed during this period.^13^ However, it remains unclear what components of Medicare spending drove changes in geographic variation and thus what policies were likely to be responsible for any geographic convergence.

## Methods

In this cross-sectional study, we evaluated the components of Medicare spending that were associated with reductions in geographic variation and potential policy mechanisms after the passage of the ACA. Because we analyzed data aggregated to the hospital referral region (HRR) level and publicly reported on the CMS website, the study was not considered human participant research according to the Regulations for the Protection of Human Subjects (45 CFR §46). The study population included all Medicare fee-for-service beneficiaries 65 years or older from January 1, 2007, through December 31, 2018. The study followed the Strengthening the Reporting of Observational Studies in Epidemiology (STROBE) reporting guideline.

### Data Sources

In February 2020, the CMS published an updated Medicare Geographic Variation Public Use File, which includes detailed data on health care expenditures for the Medicare fee-for-service population at the HRR level. This analysis used data from calendar years 2007 to 2018.^14^

### Measures

#### Health Care Expenditures per Beneficiary per Year

We classified total expenditures into 5 categories: (1) hospital inpatient services, (2) physician services, (3) hospital outpatient services, (4) postacute care, and (5) other services, including hospice, federally qualified health centers/rural health centers, outpatient dialysis facility, ambulatory surgery center, ambulance, Part B drugs, other unspecified physician, chiropractic, vision, hearing, speech, and other unclassified Part B services. For our variation analyses, we focus on the first 4 categories. We further classified expenditures for postacute care into 3 subcategories: (1) skilled nursing facility, (2) home health, and (3) inpatient rehabilitation facility and long-term care hospital.

We used the CMS’ price-standardized spending measures that eliminated variation due to geographic differences in local wages and input prices. We also inflated all expenditures to 2018 US dollars based on the Consumer Price Index released by the US Bureau of Labor Statistics.^15^

#### Geographic Variation in Health Care Expenditures

We used 2 measures to quantify variation in health care expenditures across 10 strata (deciles) of HRRs in each study year: (1) the difference between mean per-beneficiary expenditures in a particular HRR or stratum of HRRs and the national mean for that year (providing differences in absolute dollar terms), and (2) the ratio of the mean per-beneficiary expenditures in the highest stratum to the lowest stratum of HRRs for that year (providing differences in proportional terms). Calculating these measures involved the following. First, for each year from 2007 to 2018, we obtained the mean monthly per-beneficiary expenditures for each HRR as well as the mean nationally. Next, we placed the HRRs into 10 similar-sized deciles based on mean monthly per-beneficiary expenditures (totaling expenditures across service types). In any given year, decile 1 included HRRs with the lowest per-beneficiary total health care expenditures (the lowest stratum), whereas decile 10 included HRRs with the highest per-beneficiary total health care expenditures (the highest stratum). We then use the decile of total expenditures to calculate intradecile means of each of the 4 categories of services and the 3 subcategories of postacute care services.

### Statistical Analysis

#### Health Care Expenditures by Category of Service

We performed descriptive analyses of total health care expenditures in 2007 and 2018. These analyses included examining the proportion of expenditures in each of all 5 categories (including other services) in each year.

#### Difference Between Expenditures in Each HRR and National Mean

We sorted the HRRs into (10) deciles of per-beneficiary expenditures separately for 2007 and 2018. We then graphed the difference between national mean per-beneficiary expenditure and the mean expenditures in each decile by year, sorted by decile. We did this for total expenditures as well as the 4 categories of expenditures (in each case, sorting by the decile of total expenditures).

#### Ratio of Expenditures in Highest to Lowest Decile

First, we performed a descriptive analysis of the ratio of total expenditures and specific expenditure categories in the highest to lowest decile of HRR in each year from 2007 to 2018, graphing the results. Similarly, we examined ratios of the 9th and 8th deciles relative to the lowest deciles from 2007 to 2018 to identify whether reductions in variation were concentrated in top decile markets or occurring more uniformly across higher-spending markets (deciles 8 and 9). Second, we compared the ratios of the highest to the lowest deciles in 2007 with the ratios of the highest to lowest deciles in 2018 for total per-beneficiary expenditures, expenditures in each of the 4 categories of services, and per-beneficiary expenditures for each of the 3 subcategories of postacute care services. Specifically, we used *z* tests to test the null hypothesis that the ratios were the same in 2007 and 2018. We performed similar analyses comparing the ratios of decile 9 to decile 1 with those of decile 8 to decile 1 during this period.

#### Potential Mechanisms for Reductions in Variation

We performed a descriptive analysis to identify potential mechanisms for relative reductions in spending in regions with high vs low per-beneficiary Medicare spending. First, we identified the occurrence of federal home health antifraud enforcement interventions targeting HRRs in the top decile of per-beneficiary Medicare spending vs other deciles, including home health moratoria and interagency strike force teams, based on publicly available reports released by the Department of Justice and the Office of the Inspector General.^10^ Next, we examined changes in Medicare Advantage enrollment across deciles, noting that higher Medicare Advantage enrollment in a given region has been linked to practice-style spillovers and decreased use of health care services in traditional Medicare.^16^

We performed data analyses from July 22, 2019, to October 21, 2021, using STATA, version 16 (StataCorp LLC). Two-sided *P* < .05 indicated statistical significance.

## Results

There were 27.2 million fee-for-service Medicare beneficiaries in 2007, increasing to 28.3 million in 2018. The mean (SD) beneficiary age was 76.1 (0.8) years in 2007, decreasing to 75.3 (0.7) years in 2018 (eTable 1 in the Supplement). Women constituted 58.0% and men 42.0% of beneficiaries in 2007, changing to 55.9% and 44.1%, respectively, in 2018. Per-beneficiary Medicare spending was $9691 in 2007 and $9847 in 2018.

### Health Care Expenditures by Category of Service

**Figure 1.**
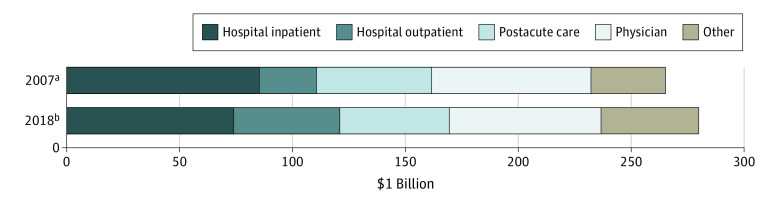
Health Care Expenditures in the Medicare Fee-for-Service System All expenditures are price standardized to eliminate spending variation due to different local wages and input prices. All expenditures are inflated to 2018 US dollars based on the Consumer Price Index released by the US Bureau of Labor Statistics. Other services include hospice, federally qualified health centers/rural health centers, outpatient dialysis facilities, ambulatory surgery centers, ambulance, Part B drugs, other unspecified physicians, chiropractic, vision, hearing, speech, and other unclassified Part B services. ^a^Total spending: $265.17 billion. ^b^Total spending: $279.93 billion.

In constant 2018 US dollars, total Medicare fee-for-service expenditures grew from $265.2 billion in 2007 to $279.9 billion in 2018. Hospital inpatient services accounted for the largest portion of total expenditures in both years, although its share of total expenditures decreased from 32% to 26%. The proportion of total expenditures that accounted for physician services decreased from 27% to 24%, and the proportion of expenditures for postacute care decreased from 19% to 17%. In contrast, the proportion of total expenditures accounted for by hospital outpatient services increased from 10% to 17%. The proportion of expenditures on other services increased as well, from 12% to 15% (Figure 1).

**Figure 2.**
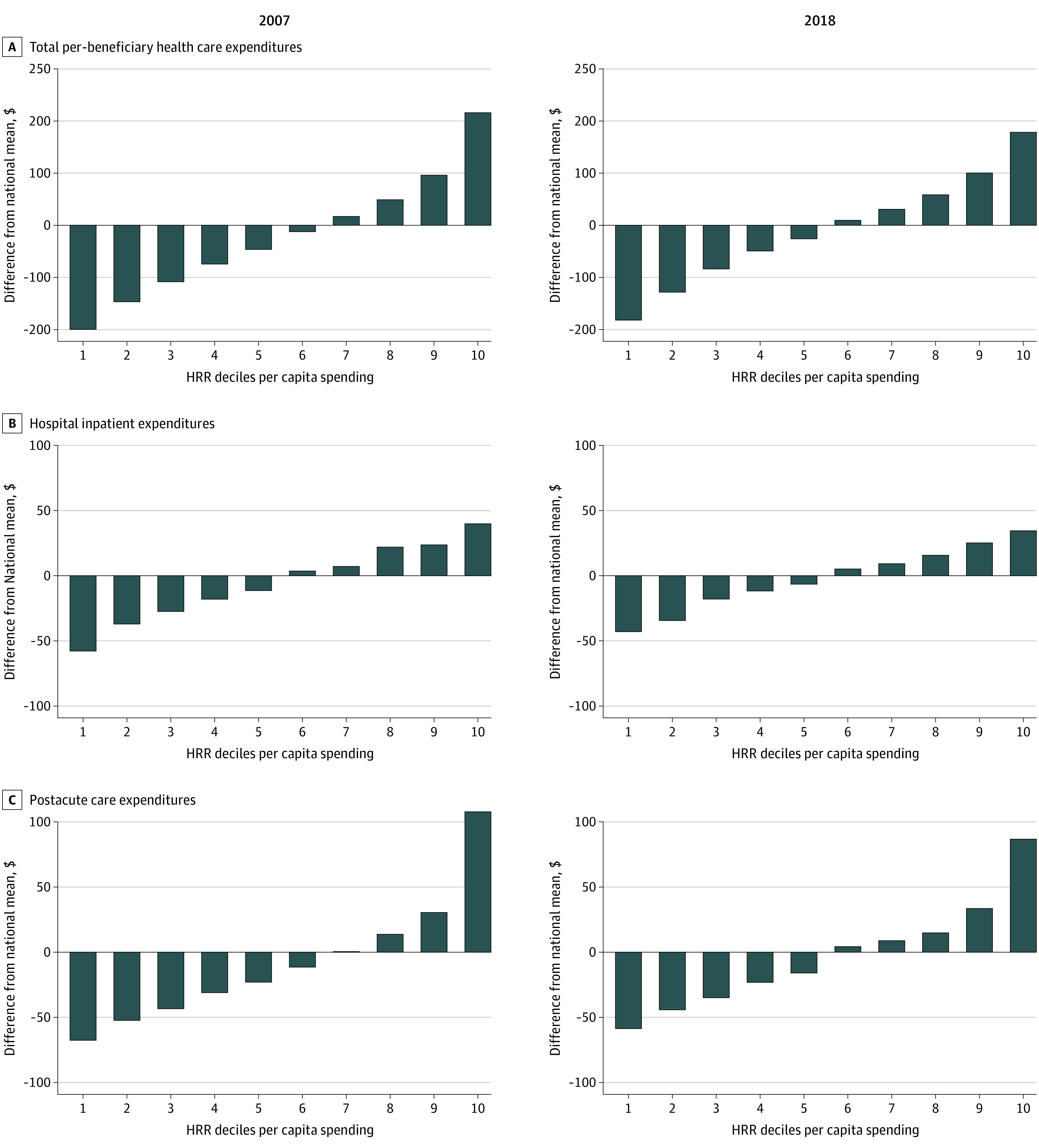
Difference Between Per-Beneficiary Monthly Health Care Expenditures in Each Decile of Hospital Referral Regions (HRRs) and the National Mean All expenditures are price standardized to eliminate spending variation due to different local wages and input prices. All expenditures are inflated to 2018 US dollars based on the Consumer Price Index released by the US Bureau of Labor Statistics. Hospital referral regions are grouped into 10 similar groups based on total per-beneficiary health care expenditures. Decile 1 is the lowest-spending group; decile 10, the highest-spending group.

Figure 2 shows the difference between mean expenditures in each decile of HRRs and the national mean, sorted by decile order, in 2007 and 2018. In 2007, the total monthly per-beneficiary expenditures in the highest-spending decile was $215 higher than the national mean. In 2018, it was $178 higher than the national mean. Conversely, in 2007, the total monthly per-beneficiary expenditures in the lowest-spending decile was $200 lower than the national mean. In 2018, it was $182 lower than the national mean. In other words, per-beneficiary monthly expenditures were $415 higher in the top vs bottom decile in 2007. By 2018, geographic variation in spending had reduced, and per-beneficiary monthly expenditures were $361 higher in the top vs bottom decile. Focusing on specific spending categories, we found reductions in geographic variation in spending for postacute care spending: monthly postacute care spending for the highest decile decreased from $108 above the national mean in 2007 to $86 above the national mean in 2018. There was less change for lower-spending HRRs: the lowest-spending decile was $68 below the national mean in 2007 and $59 below the national mean in 2018. Other spending categories showed little change by decile (eFigure 1 in the Supplement).

**Figure 3.**
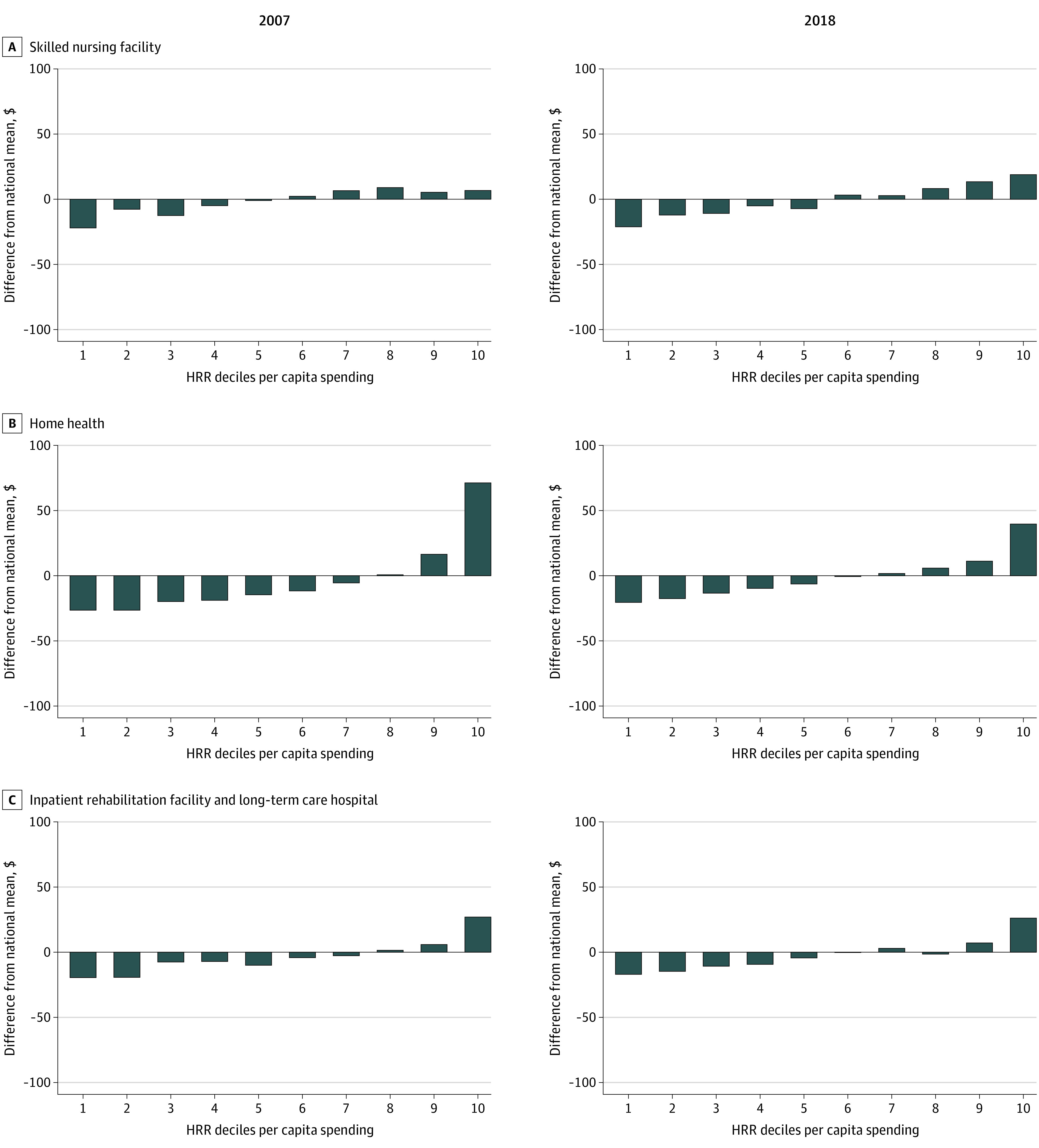
Difference in Medicare Postacute Care Per Capita Monthly Spending and National Mean by Hospital Referral Region (HRR) Deciles and Service Type All expenditures are price standardized to eliminate spending variation due to different local wages and input prices. All expenditures are inflated to 2018 US dollars based on the Consumer Price Index released by the US Bureau of Labor Statistics. The HRRs are grouped in similar deciles based on total per-beneficiary health care expenditures. Decile 1 is the lowest-spending group; decile 10, the highest-spending group.

We further divided postacute care expenditures into subcategories of skilled nursing facility, home health, and inpatient rehabilitation facility and long-term care hospital (the latter 2 subcategories were combined due to their low expenditure levels). The results show a decline in geographic variation in expenditures on home health care but little decline for other categories (Figure 3). For example, the total monthly per-beneficiary home health expenditures in the highest-spending HRRs was $71 higher than the national average in 2007 but only $40 higher than the national average in 2018.

### Ratio of Expenditures in Highest to Lowest Decile

**Figure 4.**
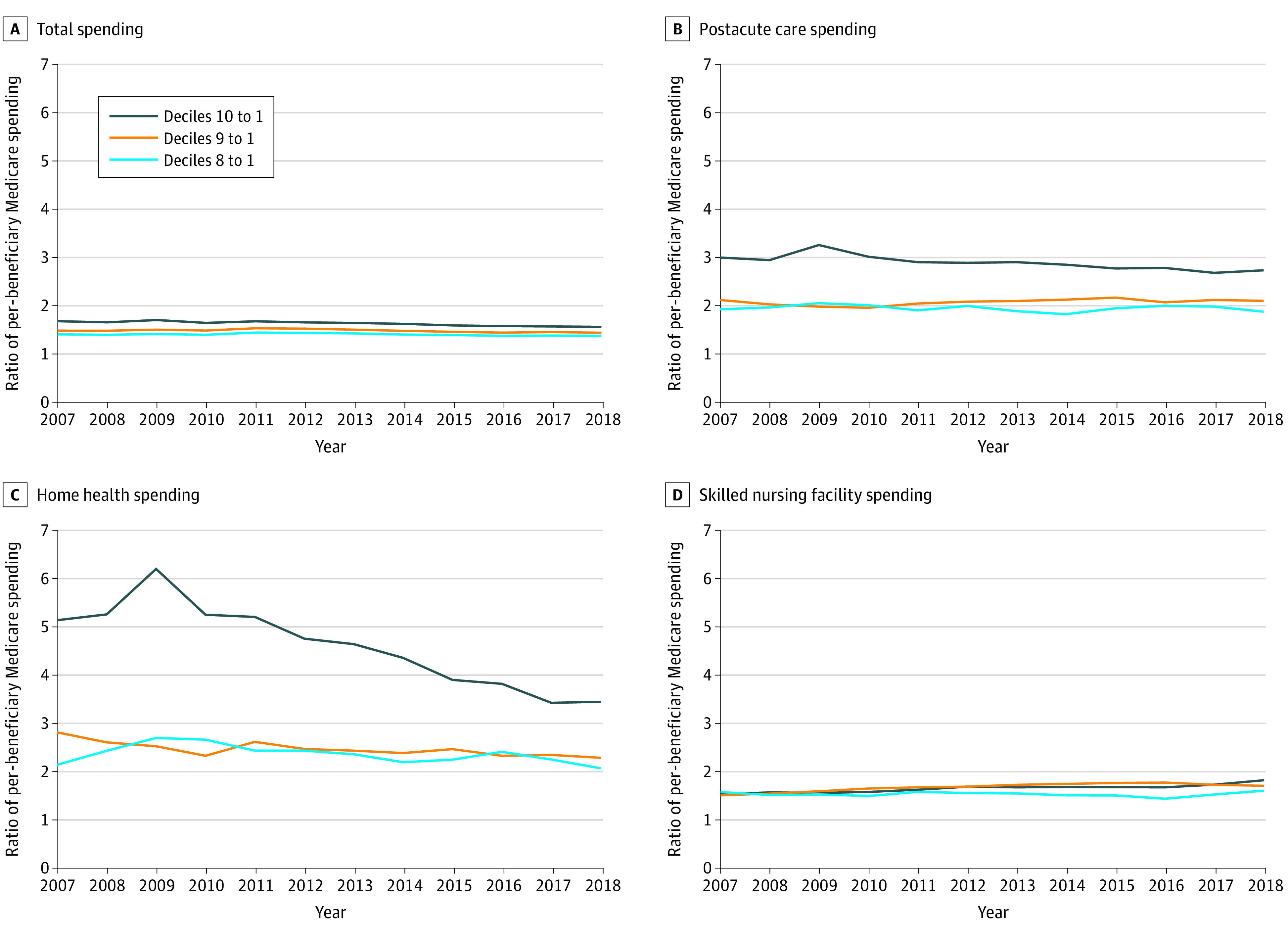
Ratio of Per-Beneficiary Medicare Spending by Total Spending Decile for Selected Categories All expenditures are price standardized to eliminate spending variation due to different local wages and input prices. All expenditures are inflated to 2018 US dollars based on the Consumer Price Index released by the US Bureau of Labor Statistics. Hospital referral regions are grouped in similar deciles based on total per-beneficiary health care expenditures. Decile 1 is the lowest-spending group; decile 10, the highest-spending group. The pronounced spike in home health spending in decile 10 to decile 1 in 2009 is driven by high per capita expenditures in Miami, Florida; McAllen, Texas; and Harlingen, Texas.

Geographic variation in total per-beneficiary health care expenditures, as measured by the ratio of per-beneficiary health care expenditures in the highest to lowest decile of HRRs, was generally stable from 2007 to 2011 and then declined steadily from 2012 to 2018 (Figure 4A). Notably, we observed a similar pattern using the coefficient of variation as a measure of variation (eFigure 2 in the Supplement).

The ratio of total per-beneficiary health care expenditures in the top decile to the bottom decile of HRRs was 1.68 in 2007 (95% CI, 1.57 to 1.79) vs 1.56 in 2018 (95% CI, 1.52 to 1.61), a difference of −0.12 (95% CI, −0.21 to −0.02; *P* = .01) (Table and eTable 2 in the Supplement). Examining the 4 leading categories of expenditures revealed that geographic variation declined the most for postacute care, with a difference between the ratios in 2007 and 2018 of −0.26, although the change was statistically insignificant (95% CI, −0.75 to 0.23; *P* = .30) (Table and Figure 4B). Within postacute care services, there was a substantial decline in geographic variation of the ratio of top to bottom deciles for home health services from 5.14 in 2007 to 3.45 in 2018, with the difference between the ratios in 2007 and 2018 of −1.69 (95% CI, −3.30 to −0.09; *P* = .04) (Table and Figure 4C). Geographic variation for skilled nursing facility services increased (0.29 [95% CI, 0.05-0.52]; *P* = .02) (Table and Figure 4D). There was no change in geographic variation in expenditures for hospital inpatient (−0.04 [95% CI, −0.16 to 0.08]; *P* = .54), hospital outpatient (−0.09 [95% CI, −0.21 to 0.04]; *P* = .17), or physician services (0.10 [95% CI, −0.07 to 0.26]; *P* = .25). Across spending categories, there was little or no reduction in the ratios of the 9th and 8th spending deciles to the lowest-spending deciles (Figure 4 and Table). For example, for home health, the change in the ratio of the 9th to the lowest-spending decile from 2007 to 2018 was smaller and statistically insignificant (−0.53 [95% CI, −1.11 to 0.05]; *P* = .08), and the change was even more attenuated for the ratio of the 8th to the lowest-spending decile (−0.07 [95% CI, −0.49 to 0.34]; *P* = .73). These results imply that reductions in variation were concentrated among markets in the highest-spending decile.

**Table.** Ratio of Per-Beneficiary Health Care Expenditures Across Deciles of HRRs for 2007 vs 2018

Medicare fee-for-service per-beneficiary expenditures^a^	Ratio of higher to lower decile		Difference (95% CI)	*P* value for difference^b^
	2007	2018		
**Tenth to first decile spending by category**				
Total	1.68	1.56	−0.12 (−0.21 to −0.02)	.01
Hospital				
Inpatient	1.48	1.44	−0.04 (−0.16 to 0.08)	.54
Outpatient	0.99	0.91	−0.09 (−0.21 to 0.04)	.17
Physician services	1.57	1.66	0.10 (−0.07 to 0.26)	.25
Postacute care	3.00	2.74	−0.26 (−0.75 to 0.23)	.30
SNFs	1.53	1.82	0.29 (0.05 to 0.52)	.02
Home health	5.14	3.45	−1.69 (−3.30 to −0.09)	.04
IRFs and LTCHs	4.62	4.46	−0.15 (−2.02 to 1.71)	.87
**Ninth to first decile spending by category**				
Total	1.48	1.44	−0.04 (−0.07 to −0.02)	.002
Hospital				
Inpatient	1.40	1.39	−0.01 (−0.08 to 0.05)	.71
Outpatient	0.86	0.92	0.06 (−0.05 to 0.17)	.27
Physician services	1.56	1.66	0.10 (−0.06 to 0.26)	.24
Postacute care	2.12	2.10	−0.02 (−0.24 to 0.20)	.89
SNFs	1.51	1.71	0.20 (−0.01 to 0.41)	.06
Home health	2.81	2.29	−0.53 (−1.11 to 0.05)	.08
IRFs and LTCHs	2.98	2.93	−0.05 (−1.38 to 1.29)	.95
**Eighth to first decile spending by category**				
Total	1.41	1.38	−0.03 (−0.06 to −0.01)	.01
Hospital				
Inpatient	1.39	1.34	−0.06 (−0.12 to 0.00)	.05
Outpatient	1.01	0.95	−0.06 (−0.24 to 0.11)	.49
Physician services	1.39	1.53	0.14 (−0.06 to 0.34)	.17
Postacute care	1.93	1.88	−0.05 (−0.27 to 0.17)	.65
SNFs	1.58	1.60	0.03 (−0.18 to 0.24)	.78
Home health	2.14	2.07	−0.07 (−0.49 to 0.34)	.73
IRFs and LTCHs	2.64	2.24	−0.39 (−1.40 to 0.61)	.44

Abbreviations: FFS, fee-for-service; HRRs, hospital referral regions; IRF, inpatient rehabilitation facility; LTCH, long-term care hospital; SNF, skilled nursing facility.

^a^ All expenditures are price standardized to eliminate spending variation due to different local wages and input prices. All expenditures are inflated to 2018 US dollars based on the Consumer Price Index released by the US Bureau of Labor Statistics. The HRRs are grouped in similar deciles based on total per-beneficiary health care expenditures. Decile 1 is the lowest-spending group; decile 10, the highest-spending group.

^b^ Calculated using standard errors clustered at the HRR level. Medicare FFS population within an HRR is used as the weight.

### Potential Mechanisms for Reduced Variation

We identified 39 HRRs that were in the top per-beneficiary spending decile in either 2007 or 2018 or in both years. The 39 high-spending regions were concentrated in the South, with 11 in Texas, 6 in Florida, and 9 in Louisiana. Every geographic area targeted by the CMS home health new provider moratoria during this period was in the top decile of Medicare spending in 2007 (Dallas, Texas) or both 2007 and 2018 (Fort Lauderdale and Miami, Florida; Chicago, Illinois; Detroit, Michigan; and Houston, Texas) (eTable 3 in the Supplement). Of the 15 HRRs that were targeted by interagency strike force teams, 9 were also part of the top per-beneficiary spending decile (eTable 4 in the Supplement). Moreover, 5 of the remaining 6 markets with home health antifraud enforcement were in Florida or Texas, proximate or adjacent to the highest-spending HRRs. In summary, home health antifraud enforcement activities were highly concentrated in the top-spending HRRs where home health spending fell significantly relative to lower-spending regions.

We plotted Medicare Advantage penetration across per-beneficiary spending deciles from 2007 to 2018 (eFigure 3 in the Supplement). Medicare Advantage penetration in the highest-spending decile was 10 percentage points higher than in the lowest-spending decile in 2018 (50% vs 40%). This pattern suggests that differential Medicare Advantage penetration was correlated with spending reductions in the highest decile relative to other deciles.

## Discussion

This analysis of geographic variation in per-beneficiary health care expenditures within the Medicare fee-for-service system has 3 notable findings. First, we observed a decline in geographic variation in total expenditures from 2007 to 2018. Whereas per-beneficiary monthly expenditures were $415 higher in the top vs bottom deciles in 2007, they were only $361 higher in 2018. Notably, we found reductions in geographic variation were concentrated in the highest-spending decile—the ratios of the 9th and 8th to the lowest-spending deciles were more constant during this period—showing that reductions in relative spending were not uniform across all higher-spending deciles but rather focused on outlier regions. Second, the decline in geographic variation appears to have begun in 2012, soon after Medicare began implementing value-based payment policies and increased antifraud enforcement after the ACA. Third, the types of services that accounted for the decrease in geographic variation were postacute care (which declined as a proportion of total expenditures), and most of the decline in geographic variation in postacute care was related to home health care. Notably, for both hospital and physician services, geographic variation was unchanged over the study period.

Historically, postacute care expenditures, and home health expenditures in particular, exhibited larger geographic variations than other categories of expenditures. For example, a 2013 report by the National Academy of Medicine found substantial geographic variation in Medicare expenditures in 2007 to 2009, and 73% of this variation was due to postacute care. In some areas, the magnitude of expenditures on postacute care was thought to be suggestive of waste if not fraud. To address the unexplained geographic variation, the National Academy recommended that Medicare payment policy reforms target clinical decision-making rather than geography—that is, value-based purchasing over reducing Medicare payments in the high-spending geographic areas.^4^ A 2017 report by the Medicare Payment Advisory Commission also documented that postacute care, and home health in particular, exhibited the greatest geographic variation in health care expenditures as of 2013 to 2014.^17^

A potential explanation for declining geographic variation in home health care expenditures is Medicare’s efforts to curb fraud, waste, and abuse. A 2019 Medicare Payment Advisory Commission report found that the volume of home health episodes grew during 2002 to 2011 and then decreased during 2012 to 2017, with the decline mostly concentrated in areas with the highest use of health services.^18^ This reduction coincided with Medicare’s efforts to reduce fraud in home health, such as banning the entry of new home health agencies in some regions and pursuing criminal investigations, as well as a 2011 Medicare policy requiring an in-person visit with a physician to approve additional episodes of home health care.^18,19^ In support of this explanation, we found that the HRRs in the highest decile of per-beneficiary Medicare spending had sizable reductions in home health spending relative to lower-spending markets and were also a key focus of home health antifraud enforcement by federal agencies.

Value-based payment reforms initiated under the ACA are also potential mechanisms for the decreased geographic variation in home health and postacute care spending. In 2012, the first accountable care organizations (ACOs) began their contracts with the CMS. Previous studies have reported an association between participation in ACOs and reduced spending on home health.^20,21^ Moreover, many of the HRRs in the highest-spending decile were located in states such as Florida, Louisiana, and Texas, where ACOs covered a relatively high percentage of Medicare beneficiaries (ie, 10% to ≥20%).^22^ Similarly, we found higher Medicare Advantage enrollment growth in the highest-spending decile relative to other regions. Previous studies have shown some evidence that regions with higher enrollment in private Medicare Advantage plans may lead to more efficient practice patterns that may spill over to traditional Medicare enrollees.^16^ Given that Medicare Advantage plans have been shown to incorporate less home health than traditional Medicare, this factor could have led to reductions in home health use.^23,24^ However, both ACOs and Medicare Advantage have also been shown to reduce use of skilled nursing facilities, which is inconsistent with our finding of increased use of skilled nursing facility care in the highest- vs lowest-spending regions.^20,21,25,26^ Other value-based payment reforms, such as bundled payment and financial penalties for hospital readmissions, had limited or no effect on home health use.^27,28,29,30^

On the other hand, ongoing payment reforms could contribute to geographic convergence in expenditures. For example, Medicare implemented a new home health payment model in 2020, which relies less on therapy provision and more on clinical characteristics.^31^ Previous studies have found that earlier Medicare payment reforms for home health initiated by the Balanced Budget Act reduced geographic variation in expenditures.^32^

Notably, our analysis did not reveal reductions in geographic variation in hospital inpatient expenditures despite the ACA implementing numerous policies targeting hospital care. Policies such as bundled payments and the Hospital Readmissions Reduction Program created incentives to improve quality of care, reduce length of stay, and prevent readmissions.^33,34^ However, none of these policies addressed the key factor driving hospital inpatient expenditures: the initial decision to hospitalize a patient.^35^ Across the US, hospitalization rates ranged from 191.9 to 272.4 per 1000 adults aged 65 to 84 years in 2016.^36^ Although ACOs did create incentives to reduce hospitalizations, researchers have found limited evidence that ACOs reduced avoidable hospitalizations.^37^

### Limitations

First, a growing proportion of Medicare beneficiaries are enrolled in Medicare Advantage, which is not included in our analysis. Second, we presented descriptive evidence and prior research findings suggesting that antifraud enforcement, more than value-based payment reforms, was responsible for reductions in geographic variation in Medicare fee-for-service spending from 2012 to 2017. However, regression analyses and a strategy to identify causal effects would be needed to distinguish which of these policies may have been responsible for reduced home health spending and geographic variation.

## Conclusions

In this cross-sectional study of US Medicare enrollees, geographic variation in per-beneficiary health care expenditures in fee-for-service Medicare declined from 2007 to 2018. This reduction coincided with implementation of the ACA, and the services for which geographic variation declined the most were targeted by specific ACA and Medicare antifraud initiatives. However, substantial geographic variation remains, suggesting that further efforts will be needed to identify and reduce the portion of geographic variation in health care expenditures that represents unnecessary or wasteful services.

## Notes

Supplement. **eFigure 1.** Difference Between Per-Beneficiary Monthly Health Care Expenditures in Each Decile of Hospital Referral Regions and the National Mean: Physician and Hospital Outpatient Services **eFigure 2.** Coefficient of Variation (SD Divided by Mean, %) of Medicare Fee-for-Service Per Capita Monthly Total Spending Across All HRRs, 2007-2018 **eFigure 3.** Medicare Advantage Penetration by Decile of Per-Beneficiary Medicare Spending **eTable 1.** Average Beneficiary Characteristics Across Hospital Referral Regions **eTable 2.** Ratio of Per-Beneficiary Medicare Spending by Total Spending Decile for Selected Categories **eTable 3.** Top Spending Decile Status of Hospital Referral Regions Targeted by New Provider Moratoria **eTable 4.** Top Spending Decile Status of Hospital Referral Regions Targeted by HEAT Strike Force
