# Supplementary material for: Geographic Variation in Medicare Fee-for-Service Health Care Expenditures Before and After the Passage of the Affordable Care Act
Source: JAMA Health Forum. 2021 Dec 10;2(12):e214122. doi: 10.1001/jamahealthforum.2021.4122 (PMC8796890; doi:10.1001/jamahealthforum.2021.4122)
Supplement: Supplement. — eFigure 1. Difference Between Per-Beneficiary Monthly Health Care Expenditures in Each Decile of Hospital Referral Regions and the National Mean: Physician and Hospital Outpatient Services eFigure 2. Coefficient of Variation (SD Divided by Mean, %) of Medicare Fee-for-Service Per Capita Monthly Total Spending Across All HRRs, 2007-2018 eFigure 3. Medicare Advantage Penetration by Decile of Per-Beneficiary Medicare Spending eTable 1. Average Beneficiary Characteristics Across Hospital Referral Regions eTable 2. Ratio of Per-Beneficiary Medicare Spending by Total Spending Decile for Selected Categories eTable 3. Top Spending Decile Status of Hospital Referral Regions Targeted by New Provider Moratoria eTable 4. Top Spending Decile Status of Hospital Referral Regions Targeted by HEAT Strike Force [file jamahealthforum-e214122-s001.pdf]

Supplementary Online Content

Sood N, Yang Z, Huckfeldt P, Escarce J, Popescu I, Nuckols T. Geographic variation in Medicare fee-for-service health care expenditures before and after the passage of the Affordable Care Act. *JAMA Health Forum*. 2021;2(12):e214122. doi:10.1001/jamahealthforum.2021.4122

eFigure 1. Difference Between Per-Beneficiary Monthly Health Care Expenditures in Each Decile of Hospital Referral Regions and the National Mean: Physician and Hospital Outpatient Services

eFigure 2. Coefficient of Variation (SD Divided by Mean, %) of Medicare Fee-for-Service Per Capita Monthly Total Spending Across All HRRs, 2007-2018

eFigure 3. Medicare Advantage Penetration by Decile of Per-Beneficiary Medicare Spending

eTable 1. Average Beneficiary Characteristics Across Hospital Referral Regions

eTable 2. Ratio of Per-Beneficiary Medicare Spending by Total Spending Decile for Selected Categories

eTable 3. Top Spending Decile Status of Hospital Referral Regions Targeted by New Provider Moratoria

eTable 4. Top Spending Decile Status of Hospital Referral Regions Targeted by HEAT Strike Force

This supplementary material has been provided by the authors to give readers additional information about their work.

© 2021 Sood N et al. *JAMA Health Forum.*

eFigure 1. Difference Between Per-Beneficiary Monthly Health Care Expenditures in Each Decile of Hospital Referral Regions and the National Mean: Physician and Hospital Outpatient Services

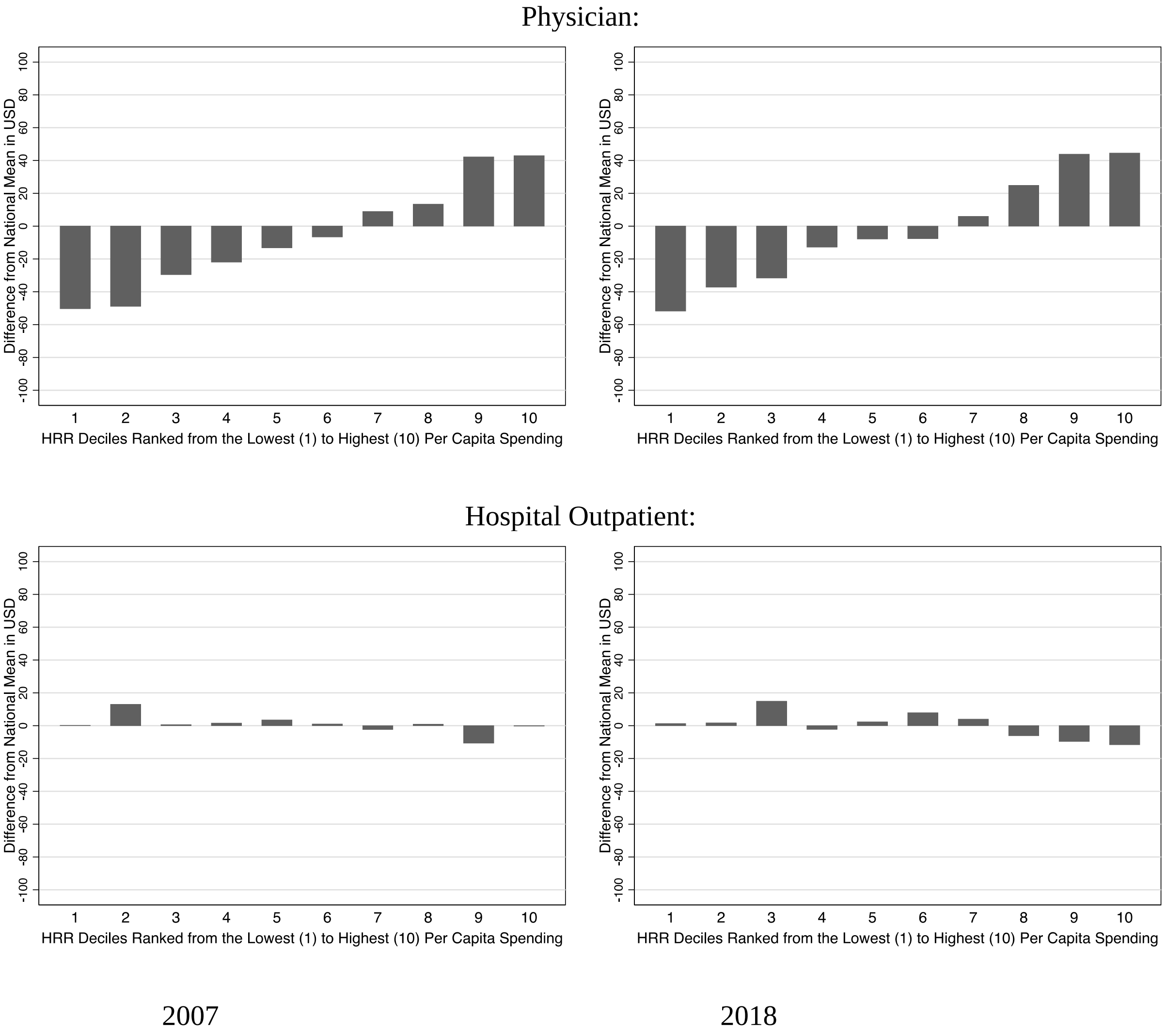

Notes:

(1) All expenditures are price-standardized to eliminate spending variation due to different local wages and input prices.

(2) All expenditures are inflated to the 2018 US dollars, based on the Consumer Price Index released by the Bureau of Labor Statistics.

(3) HHRs are grouped in to 10 similar groups based on total per-beneficiary healthcare expenditures. Decile 1 is the lowest spending group and decile 10 is the highest spending group.

© 2021 Sood N et al. *JAMA Health Forum.*

eFigure 2. Coefficient of Variation (SD Divided by Mean, %) of Medicare Fee-for-Service Per Capita Monthly Total Spending Across All HRRs, 2007-2018

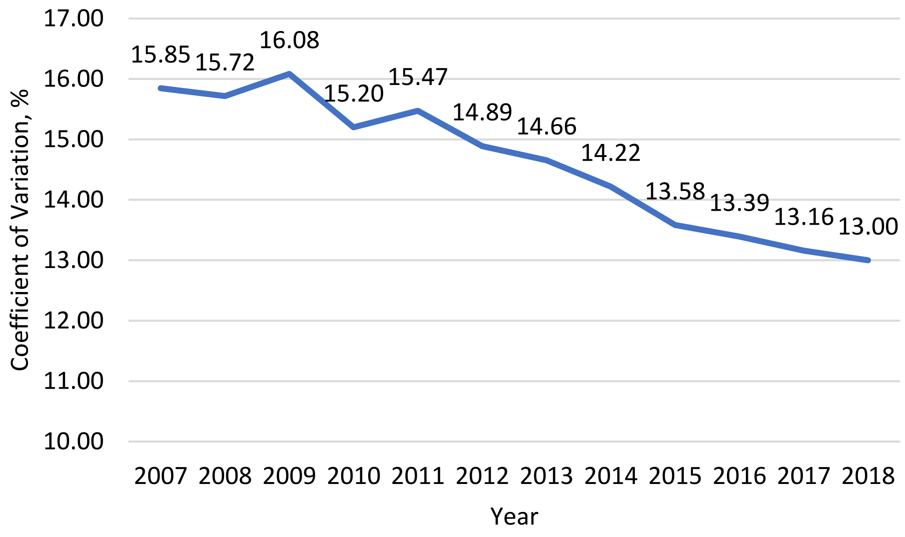

Note: All expenditures are price-standardized to eliminate spending variation due to different local wages and input prices.

© 2021 Sood N et al. *JAMA Health Forum.*

eFigure 3. Medicare Advantage Penetration by Decile of Per-Beneficiary Medicare Spending

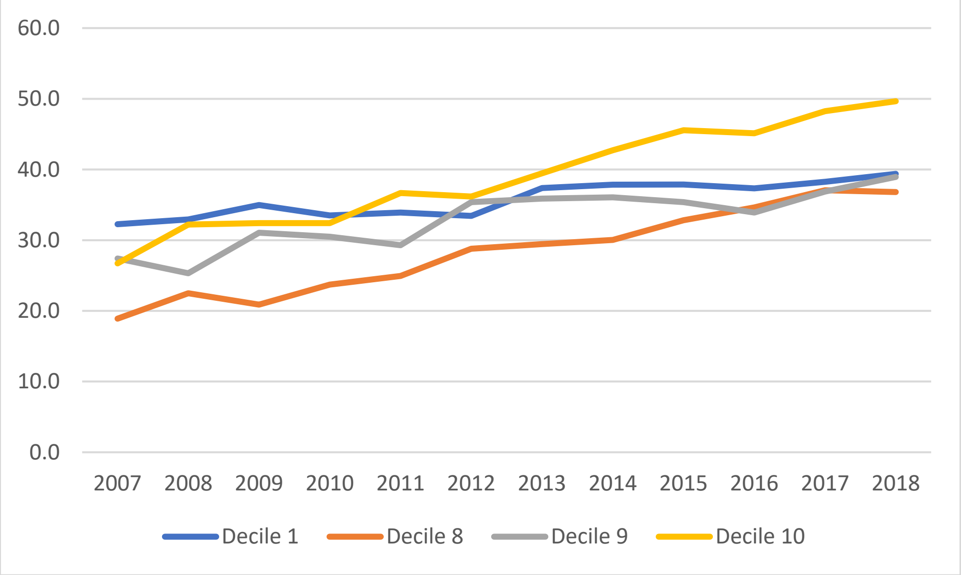

Note: MA penetration rate weighted by number of total of fee-for-service Medicare and Medicare Advantage beneficiaries in each HRR.

eTable 1. Average Beneficiary Characteristics Across Hospital Referral Regions

	2007	2018
Age, mean (SD)	76.1 (0.8)	75.3 (0.7)
% Female	58.0%	55.9%
% non-Hispanic white	85.1%	81.9%
% African American	7.4%	7.2%
% Hispanic	4.5%	5.1%
% Other/unknown race/ethnicity	3.0%	5.8%
% Eligible for Medicaid	14.4%	12.8%
Average HCC score, mean (SD)	0.98 (0.08)	0.96 (0.08)
Per beneficiary spending (2018 dollars)	9,691	9,847
Number of hospital referral regions	306	306
Number of fee-for-service Medicare beneficiaries	27,190,599	28,298,071

Note: Beneficiary characteristics weighted by number of fee-for-service Medicare beneficiaries in each HRR.

© 2021 Sood N et al. *JAMA Health Forum.*

eTable 2. Ratio of Per-Beneficiary Medicare Spending by Total Spending Decile for Selected Categories

a. Total Medicare spending

	10th to 1st decile		9th to 1st decile		8th to 1st decile	
	Ratio	95% CI	Ratio	95% CI	Ratio	95% CI
2007	1.68	(1.57, 1.79)	1.48	(1.45, 1.52)	1.41	(1.37, 1.44)
2008	1.66	(1.55, 1.76)	1.48	(1.45, 1.52)	1.40	(1.37, 1.43)
2009	1.70	(1.59, 1.82)	1.50	(1.47, 1.54)	1.41	(1.38, 1.45)
2010	1.65	(1.58, 1.72)	1.49	(1.45, 1.53)	1.40	(1.37, 1.43)
2011	1.68	(1.61, 1.75)	1.53	(1.49, 1.58)	1.44	(1.40, 1.48)
2012	1.66	(1.59, 1.72)	1.53	(1.48, 1.57)	1.44	(1.40, 1.48)
2013	1.64	(1.58, 1.71)	1.50	(1.46, 1.55)	1.43	(1.39, 1.46)
2014	1.62	(1.57, 1.68)	1.48	(1.44, 1.52)	1.40	(1.37, 1.44)
2015	1.59	(1.54, 1.64)	1.46	(1.42, 1.50)	1.39	(1.36, 1.42)
2016	1.58	(1.53, 1.62)	1.44	(1.41, 1.48)	1.38	(1.35, 1.41)
2017	1.57	(1.53, 1.62)	1.45	(1.42, 1.49)	1.38	(1.35, 1.41)
2018	1.56	(1.52, 1.61)	1.44	(1.41, 1.47)	1.38	(1.35, 1.40)

b. Post-acute care spending

	10th to 1st decile		9th to 1st decile		8th to 1st decile	
	Ratio	95% CI	Ratio	95% CI	Ratio	95% CI
2007	3.00	(2.44, 3.55)	2.12	(1.89, 2.34)	1.93	(1.70, 2.16)
2008	2.94	(2.41, 3.47)	2.03	(1.82, 2.23)	1.96	(1.73, 2.19)
2009	3.26	(2.74, 3.77)	1.98	(1.75, 2.21)	2.05	(1.84, 2.26)
2010	3.01	(2.64, 3.38)	1.96	(1.74, 2.17)	2.01	(1.81, 2.21)
2011	2.90	(2.56, 3.24)	2.04	(1.84, 2.25)	1.90	(1.70, 2.11)
2012	2.89	(2.54, 3.24)	2.08	(1.82, 2.34)	2.00	(1.82, 2.17)
2013	2.90	(2.59, 3.22)	2.10	(1.88, 2.31)	1.89	(1.72, 2.05)
2014	2.85	(2.59, 3.11)	2.12	(1.92, 2.33)	1.82	(1.66, 1.98)
2015	2.77	(2.55, 3.00)	2.17	(1.92, 2.42)	1.94	(1.79, 2.10)
2016	2.78	(2.58, 2.98)	2.07	(1.84, 2.29)	2.00	(1.85, 2.15)
2017	2.68	(2.47, 2.89)	2.12	(1.88, 2.36)	1.98	(1.82, 2.14)
2018	2.74	(2.52, 2.96)	2.10	(1.90, 2.31)	1.88	(1.75, 2.00)

c. Home health spending

	10th to 1st decile		9th to 1st decile		8th to 1st decile	
	Ratio	95% CI	Ratio	95% CI	Ratio	95% CI
2007	5.14	(3.38, 6.90)	2.81	(2.31, 3.32)	2.14	(1.73, 2.55)
2008	5.26	(3.39, 7.13)	2.61	(2.11, 3.11)	2.43	(1.92, 2.94)
2009	6.20	(4.28, 8.13)	2.53	(2.03, 3.02)	2.70	(2.12, 3.27)
2010	5.25	(3.98, 6.52)	2.33	(1.87, 2.79)	2.66	(2.06, 3.27)
2011	5.21	(3.94, 6.47)	2.62	(2.08, 3.15)	2.43	(1.98, 2.89)
2012	4.76	(3.62, 5.89)	2.47	(1.96, 2.98)	2.43	(1.98, 2.88)
2013	4.64	(3.55, 5.74)	2.43	(1.92, 2.94)	2.36	(1.95, 2.77)
2014	4.36	(3.53, 5.19)	2.39	(1.90, 2.87)	2.20	(1.82, 2.57)
2015	3.90	(3.30, 4.50)	2.46	(1.89, 3.04)	2.25	(1.88, 2.61)
2016	3.82	(3.30, 4.34)	2.33	(1.79, 2.87)	2.41	(2.03, 2.79)
2017	3.43	(2.99, 3.86)	2.35	(1.81, 2.89)	2.25	(1.89, 2.61)
2018	3.45	(2.98, 3.92)	2.29	(1.78, 2.79)	2.07	(1.78, 2.36)

© 2021 Sood N et al. *JAMA Health Forum.*

d. Skilled nursing facility spending

	10th to 1st decile		9th to 1st decile		8th to 1st decile	
	Ratio	95% CI	Ratio	95% CI	Ratio	95% CI
2007	1.53	(1.35, 1.72)	1.51	(1.34, 1.68)	1.58	(1.37, 1.78)
2008	1.57	(1.40, 1.74)	1.54	(1.37, 1.72)	1.52	(1.31, 1.72)
2009	1.56	(1.38, 1.74)	1.59	(1.42, 1.77)	1.53	(1.32, 1.73)
2010	1.58	(1.42, 1.74)	1.65	(1.48, 1.82)	1.50	(1.31, 1.68)
2011	1.63	(1.43, 1.82)	1.68	(1.48, 1.87)	1.58	(1.39, 1.78)
2012	1.69	(1.49, 1.89)	1.69	(1.48, 1.90)	1.55	(1.37, 1.74)
2013	1.68	(1.49, 1.87)	1.73	(1.56, 1.90)	1.55	(1.38, 1.72)
2014	1.68	(1.51, 1.86)	1.75	(1.58, 1.91)	1.51	(1.36, 1.67)
2015	1.68	(1.49, 1.87)	1.76	(1.61, 1.92)	1.51	(1.36, 1.66)
2016	1.68	(1.47, 1.88)	1.77	(1.61, 1.94)	1.44	(1.31, 1.57)
2017	1.73	(1.51, 1.95)	1.73	(1.55, 1.90)	1.53	(1.38, 1.67)
2018	1.82	(1.54, 2.10)	1.71	(1.52, 1.90)	1.60	(1.44, 1.77)

© 2021 Sood N et al. *JAMA Health Forum.*

eTable 3. Top Spending Decile Status of Hospital Referral Regions Targeted by New Provider Moratoria

Hospital referral region	Top spending decile?
TX - Dallas	Just 2007
FL - Fort Lauderdale	2007 and 2018
FL - Miami	2007 and 2018
IL - Chicago	2007 and 2018
MI - Detroit	2007 and 2018
TX - Houston	2007 and 2018

Note: Moratoria regions come from US Department of Health and Human Services Office of the Inspector General Data Brief "Nationwide Analysis of Common Characteristics in OIG Home Health Fraud Cases", June 2016**.** https://oig.hhs.gov/oei/reports/oei-05-16-00031.pdf

eTable 4. Top Spending Decile Status of Hospital Referral Regions Targeted by HEAT Strike Force

Hospital referral region	Top spending decile?
FL - Fort Lauderdale	2007 and 2018
FL - Miami	2007 and 2018
IL - Chicago	2007 and 2018
LA - Alexandria	2007 and 2018
TX - Corpus Christi	2007 and 2018
TX - Harlingen	2007 and 2018
TX - Houston	2007 and 2018
TX - McAllen	2007 and 2018
CA - Los Angeles	Just 2018
TX- San Antonio	Proximate to top decile
FL - Lakeland	Proximate to top decile
FL - Tampa	Proximate to top decile
FL - Ocala	Proximate to top decile
FL- Orlando	Proximate to top decile
NY – New York City	Not top decile or proximate

Note: Strike Force targets come from US Department of Health and Human Services Office of the Inspector General Data Brief "Nationwide Analysis of Common Characteristics in OIG Home Health Fraud Cases", June 2016**.** https://oig.hhs.gov/oei/reports/oei-05-16-00031.pdf

© 2021 Sood N et al. *JAMA Health Forum.*
